# Management of a solitary bone cyst using a custom-made surgical guide for a minimally invasive approach: technical note and case report

**DOI:** 10.1186/s12903-024-04308-4

**Published:** 2024-05-14

**Authors:** Maxime Delarue, Cyril Perez, Quentin Lucidarme, Fabien Bornert

**Affiliations:** 1 Faculty of Dental Surgery, University of Strasbourg, 8 Rue de Sainte Elisabeth, Strasbourg, 67000 France; 2 Oral Surgery, UF8601, University Hospital of Strasbourg, 1 Place de l’Hôpital, Strasbourg, 67000 France; 3 Dental Care Unit, UF8611, University Hospital of Strasbourg, Hôpital de Hautepierre, 1 Avenue Molière, Strasbourg, 67098 France; 4 INSERM (French National Institute of Health and Medical Research), UMR 1260, Regenerative Nanomedicine, CRBS, 1 Rue Eugène Boeckel, Strasbourg, 67000 France

**Keywords:** Solitary Bone Cyst, Guided surgery, Open-frame sleeveless guide, Computer assisted surgery, 3D printing, Jaw cyst, Bone cyst

## Abstract

**Background:**

Solitary Bone Cyst (SBC), also known as a simple bone cyst, hemorrhagic cyst, or traumatic cyst is classified by the WHO among non-odontogenic benign lesions of the jaw. The article explores the use of a static 3D-printed surgical guide to treat mandibular SBC, emphasizing a minimally surgical approach for this lesion.

**Case Presentation:**

A 20-year-old woman was referred for a persistent mandibular SBC lacuna, without specific complaints. Her medical history included a previous bone trepanation for a SBC in the same area, radiologically and surgically confirmed. X-ray assessment showed a well-defined unilocular radiolucency surrounding the root of the first left lower molar (tooth #36), measuring 10 × 10 mm. Pulp sensitivity was normal. CBCT data and STL files of dental cast were obtained preoperatively and registered. A 3D-printed surgical guide was used for minimally invasive trepanation of the buccal cortical. The simulation used a targeted endodontic microsurgery approach in order to determine axis and diameter of the trephine. Surgery was performed under local anesthesia. The guide was tooth supported integrating tubes and a fork for guiding precise trepanation. A 3.5 mm round bone window was created, leaving an empty cavity confirming SBC diagnosis and permitting bone curettage. A blood clot was obtained to promote bone healing. Complete reossification was observed after 6 months. The follow-up at 2 years confirmed a complete bone healing with normal pulp sensitivity.

**Discussion:**

The 3D-printed windowed surgical guide with dental support offers big advantages, including improved visibility and reduced errors. Compared to traditional guides, it eliminates visual hindrance and allows easier and quick access to confined areas as well as an improved irrigation during drilling process. The article also highlights the importance of preoperative planning while acknowledging potential limitations and errors and surgical complications.

**Conclusion:**

The use of the 3D-printed surgical guide could be used in routine for minimally invasive intervention of SBC. This case also demonstrates the potential utility of this approach in various procedures in oral and maxillofacial surgery. The technique provides precise localization, reducing complications and enhances operative efficiency.

## Introduction

Solitary Bone Cyst (SBC), also known as a simple bone cyst, hemorrhagic cyst, or traumatic cyst is classified by the WHO among the benign lesions of the maxillae [1]. The variety of these names to describe a single lesion reflects the poor understanding of its etiopathogenesis. However, trauma seems to be one of the causes attributed to the occurrence of SBCs [2]. 

SBC is actually a pseudo-cyst. The bone cavity is not lined by an epithelial membrane. The intralesional surgical observation reveals a bony cavity containing a characteristic serous or sero-hematic fluid. The absence of a cystic wall and the intralesional fluid content confirm this diagnosis [3]. Histologically, if the operator sends a curettage product of the bony wall, one may find a very thin layer of nonspecific loose connective tissue [2]. In all cases, for SBCs, the definitive diagnosis relies on the intraoperative clinical view.

There appears to be no gender predilection, but the prevalence is higher in children and young adults around the 2nd and 3rd decades. It is a bony defect found in the metaphysis of long bones as well as in the jaws. It represents approximately 1% of maxillary cystic lesions. Furthermore, there is a strong predilection for the mandible, with over 90% of lesions found there [4, 5]. 

80% of SBCs are asymptomatic and discovered incidentally on X-rays. The remaining cases may be revealed by dental sensitivities, bone pain, intra-oral swelling or facial asymmetry [6, 7]. 

The SBC appears on standard imaging exams (X-rays and CBCT) as a mostly unilocular radiolucency but may sometimes exhibit intralesional septa. It can erode the bony cortices and is bordered by a rim of peripheral osteosclerosis indicating its chronic evolution and benign nature. The buccal and lingual cortices are blown out, and the inferior alveolar nerve canal is indiscernible radiographically inside this lesion [3]. 

A surgical approach is indicated to achieve a complete bone healing without recurrence. This window can be done using microsurgical instruments mounted on a handpiece, such as a micro-saw or other dedicated bone drills or by using piezo-surgery. Once again, the diagnosis is confirmed intraoperatively upon breaching this bony defect. The goal of surgery is to provide “decompression” of the bony cavity and to perform a proper curettage of the walls to stimulate a bleeding to fill the cavity by a blood clot. This will induce pro-osteogenic action of the adjacent bony walls. However, follow-up is necessary to confirm the complete reossification of the bony defect [5]. 

Understanding the importance of precise preoperative diagnosis is crucial to optimizing the surgical approach, remaining minimally invasive for bone, teeth vitality, roots and inferior alveolar bundle nerve preservation, reducing operative time, and optimizing bone healing.

In the present report, we introduce a custom-made 3D-printed surgical guide allowing accurate access of SBC via a minimally invasive surgery inspired by a targeted endodontic microsurgery approach [8]. 

## Case presentation

A 20-year-old woman was referred by her dentist concerning a persistent mandibular radiolucency to the Department of Oral Surgery, University Hospital of Strasbourg. She did not have any complaints. His medical history revealed a previous bone trepanation six years ago in the same mandibular area for a SBC. A preliminary diagnosis was previously established through Cone Beam Computed Tomography (CBCT) and Magnetic Resonance Imaging (MRI) assessment [9]. Intraoperatively, the diagnosis was clinically confirmed by the presence of an empty bony cavity, as widely documented in literature [3, 10]. 

**Fig. 1:**
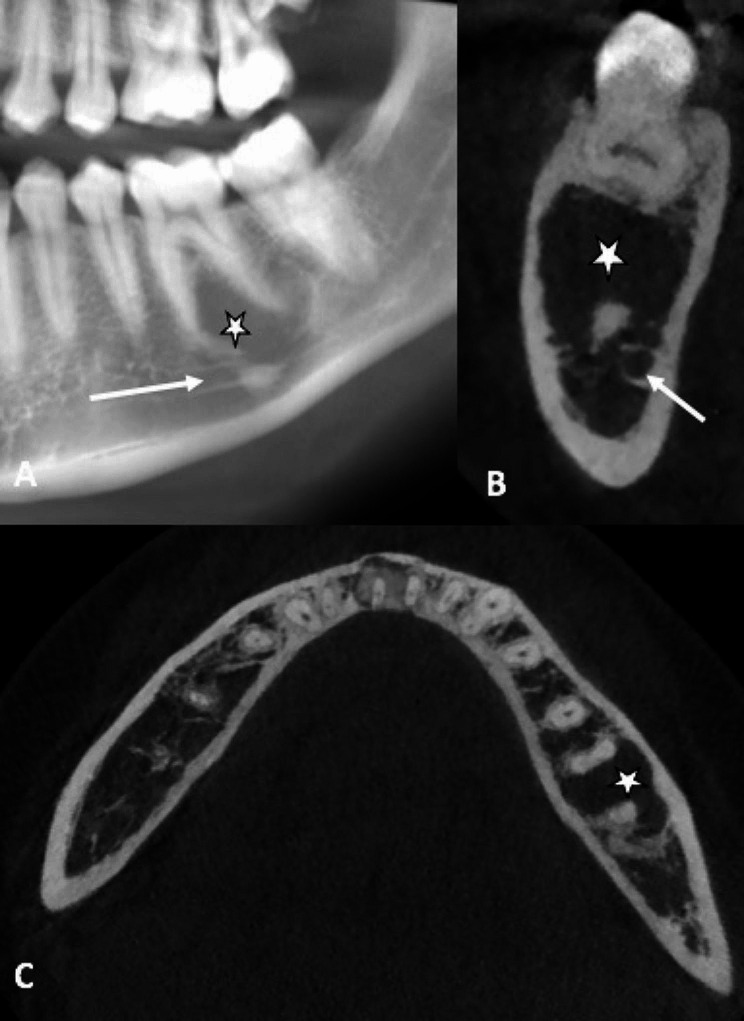
CBCT assessment. A. Reconstructed panoramic view. B. Orthoradial slice of mandible through tooth 36. C. Axial slice. Legend: These views show radiolucent lacuna surrounding roots of tooth #36 (white star*) (white arrow shows mandibular canal)

The new CBCT showed a well-defined unilocular radiolucent area below the root apices of the left mandibular first molar, measuring 10 × 10 mm (Fig. 1A, B and C) with a close proximity with mandibular canal located below. The pulp sensitivity was normal for teeth 36 and 37. Based on medical history, imaging assessment, diagnosis of persistent SBC was established. A surgical exploration was proposed to confirm diagnosis and performing a second curettage. As roots were included in the lesion and inferior alveolar canal was close to the bottom of the lesion, a surgical guide was proposed to perform a minimally invasive cortical trepanation and prevent damage to the nearby noble structures.

**Fig. 2:**
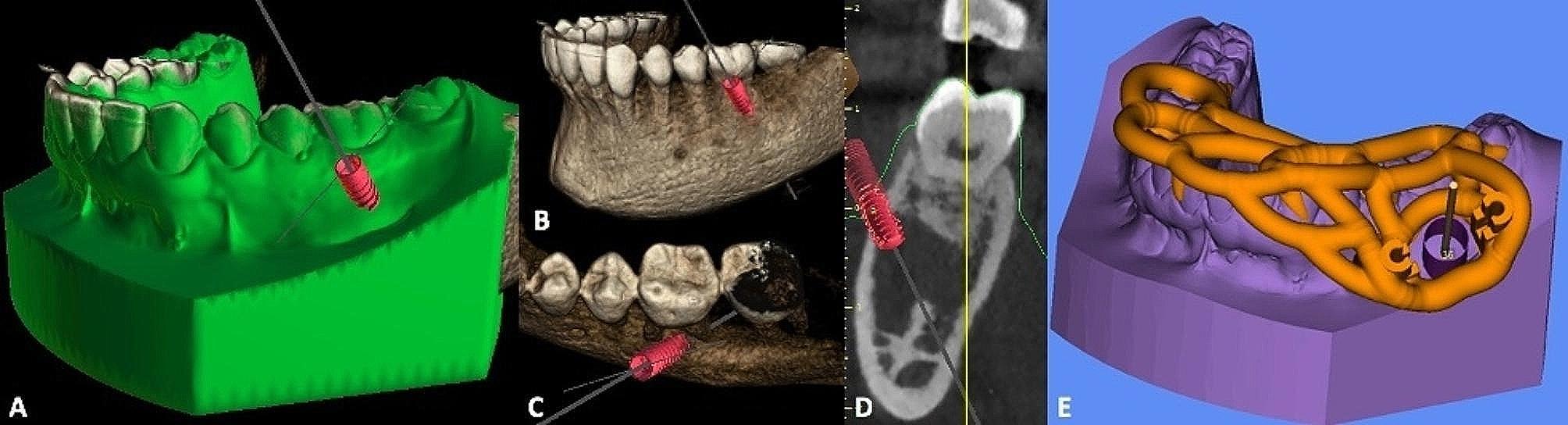
Surgical 3D planification. A. Overview of 3D images reconstructions of CBCT and mouth cast registration with simulation of implant placement. B, C, D. CBCT 3D reconstruction images showing simulation of implant placement with its apex going through buccal cortical in interradicular space of tooth #36. E. Computer aided design of custom-made surgical guide

**Fig. 3:**
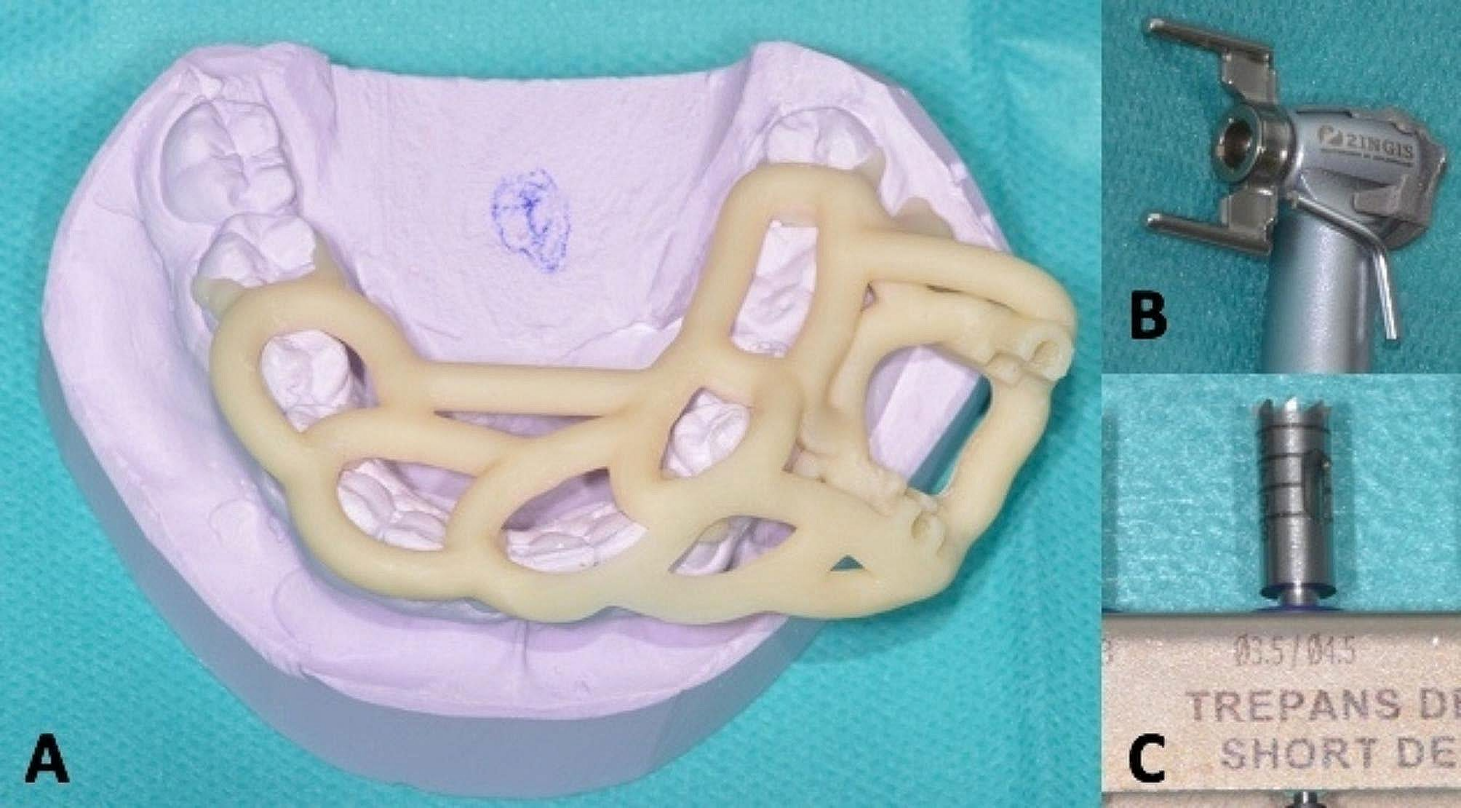
View of custom-made 3D-printed sleeveless surgical guide with guided head of the contra-angle. A. Tooth support resin surgical guide with two parallel solid tubes integrated in the framework of the guide. B. Two legs of a fork attached to the head of the contra-angle. C. Graduated short trephine Ø3.5

CBCT DICOM files dataset and stereolithographic (STL) file of dental model scans (Trios; 3Shape, Copenhagen, Denmark) were acquired preoperatively. All dataset were registered in Blue Sky Plan software (Blue Sky Bio LLC, Grayslake, IL, USA). In this case, the simulation was performed with a dental implant compatible with the diameter and length of the trephine (Fig. 2A, B, C AND D). After planning of the position, diameter, depth, axis and size of short dental trephine by the clinician, the company manufactured and shipped the individual patient surgical guide (2INGIS®, Zaventem, Belgium) (Fig. 2E). The surgical guide was printed in try-in resin using a 3D printer NextDent™ 5100 (3D Systems®). A tooth support structure was designed. It consisted of the two parallel solid tubes integrated in the framework of the guide (Fig. 3A), into which the two legs of a fork attached to the head of the contra-angle can slide (Fig. 3B). The penetration of the trephine (Fig. 3C) was controlled by the depth stop on the fork on the guide framework.

**Fig. 4:**
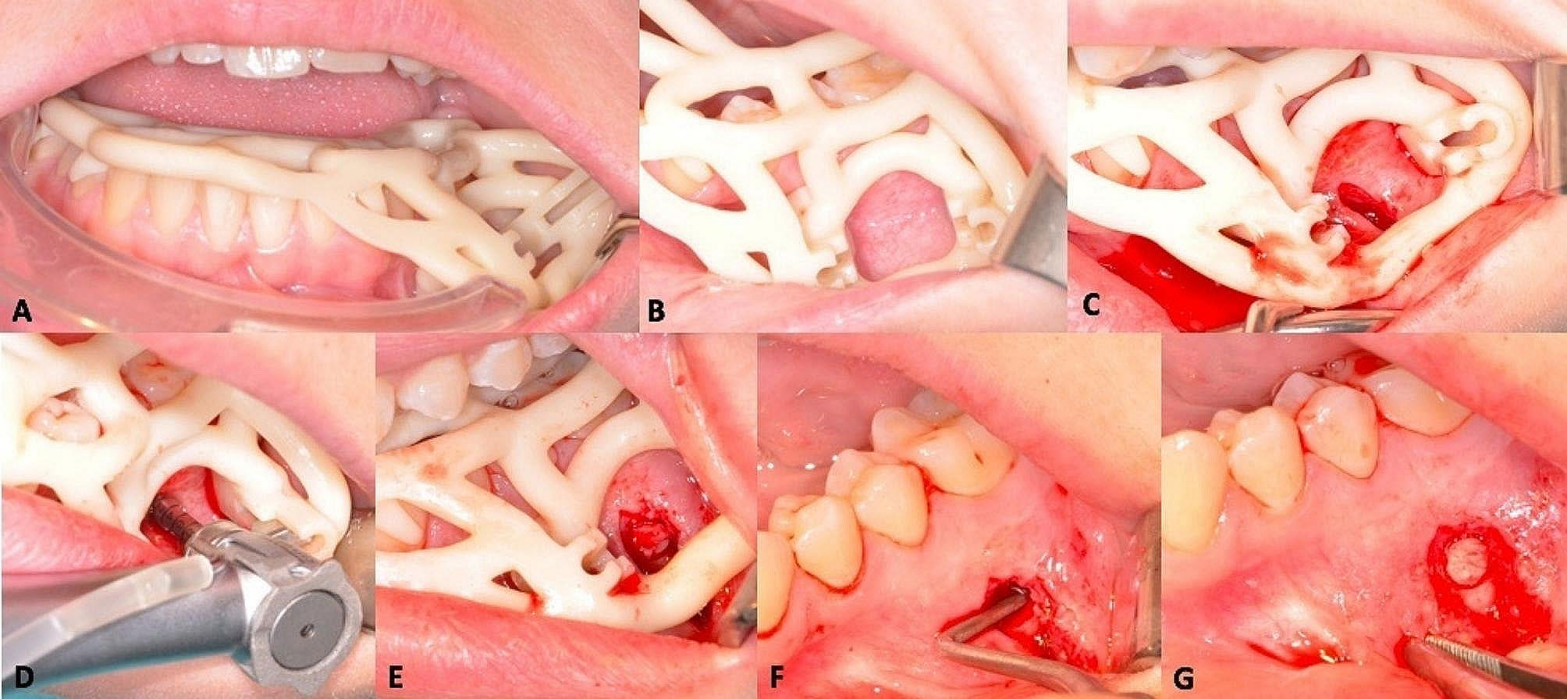
Clinical views of surgical steps procedure. A. Intraoral placement of the surgical Guide. B. Good visibility of the surgical site. C. Vertical incision in the alveolar mucosa. D. Trephine penetration in cortical bone, the head of the contra-angle is guided by the the forks insered in two parallel solid tubes integrated in the framework of the guide. E. A ring bone was removed at the point of impact on the vestibular cortical bone. F. Curettage performed using a long and thin curette to create a blood clot, ensuring bone healing. G. Repositioning of the bone lid

Local anaesthesia of buccal alveolar mucosa below the first lower molar was done. Then, the surgical guide was positioned in the mouth ((Fig. 4A and B). A vertical incision was made on the alveolar mucosa (Fig. 4C). A full-thickness flap was raised to access to the bone cortical with the trephine (Fig. 4D). A specific surgical kit was used. It included a contra-angle with guide forks of different lengths (depending on the patient’s capacity to open the mouth and laxity of cheek musculature). The guidance system was supported by a double tube (one on either side of the drilling axis). A ring of uniform thickness was created at the point of impact on the buccal cortical with the graduated short trephine of 3.5 mm diameter (Thomas; FFDM Tivoly, Bourges, France) (Fig. 4E). This “window” was removed and placed in saline solution. An empty cavity was visually and tactilely identified during slight curettage avoiding root. Diagnosis of solitary bone cyst was confirmed. The cavity’s walls were curetted until the cavity was fully filled by a blood clot (Fig. 4F). The bone lid was repositioned into the defect (Fig. 4G), and two resorbable sutures were made to close mucosa. Post-operative medications were mouthwash (chlorhexidine 0.12%) one day after surgery for seven days.

**Fig. 5:**
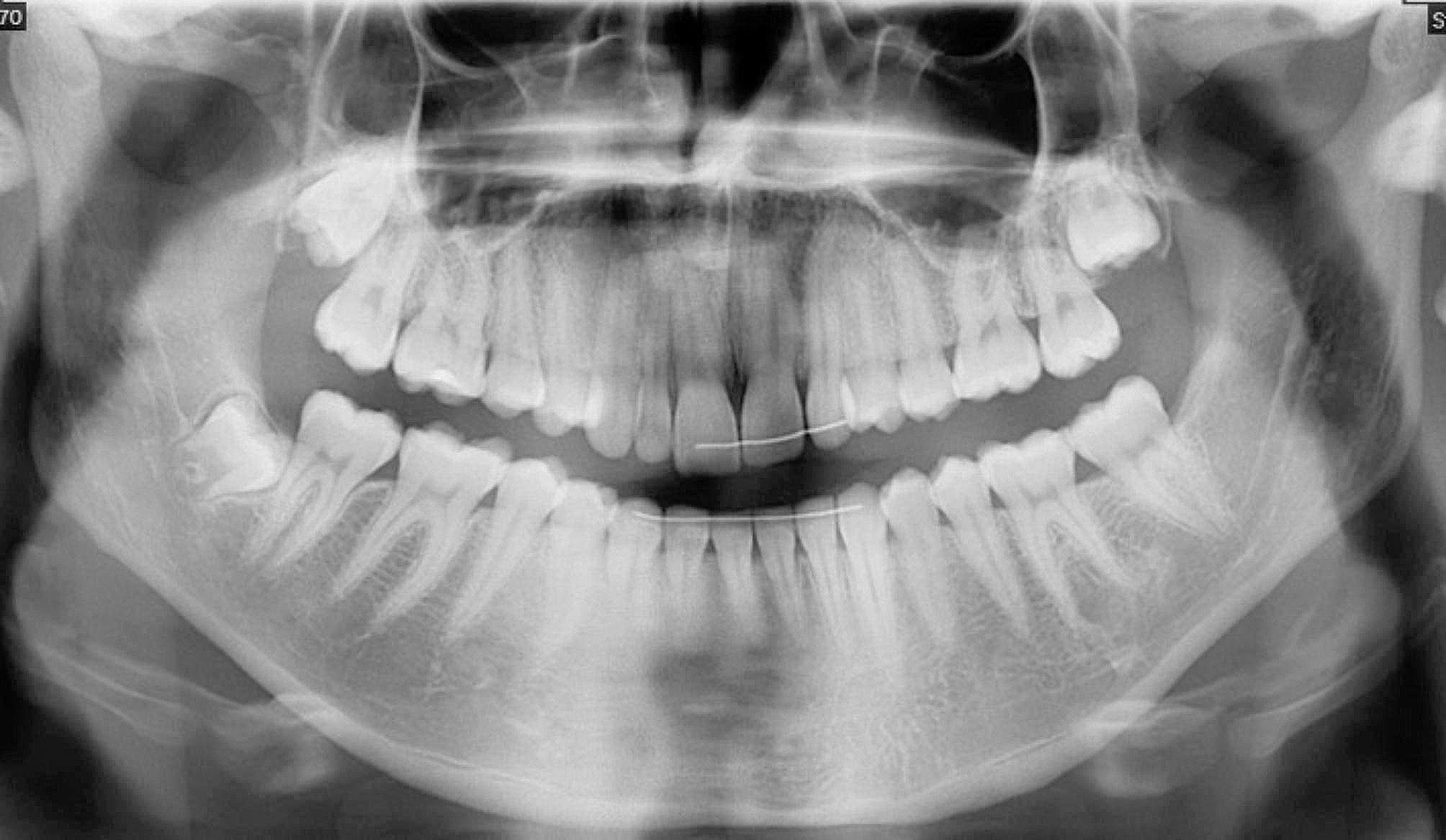
Panoramic X-ray at 6 months of follow-up. Legend: This view’s showing complete reossification of the bony defect around root of tooth #36

**Fig. 6:**
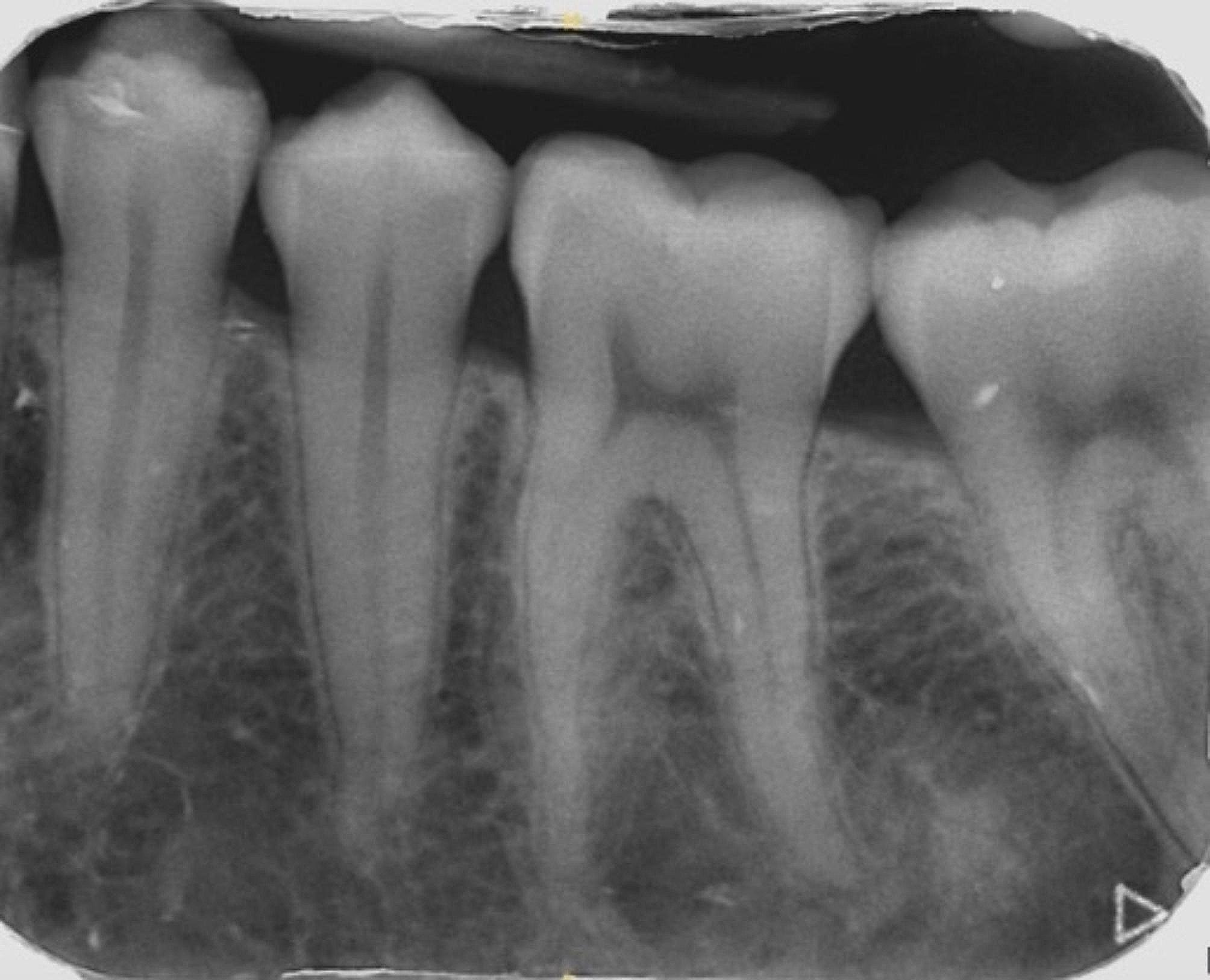
Retroalveolar X-ray of tooth #36 at 2 years of follow-up. Legend: This view’s showing sustainble complete reossification of the bony defect around root of tooth #36. A slight resorption a mesial root is also observed

The patient had only taken acetaminophen 1 g in postoperative time surgery. Surgical outcomes were uneventful. Soft tissue healing was complete after two weeks. After 6 months of follow-up a complete reossification of the bony defect was objectived (Fig. 5) and left mandibular molars had normal pulp sensitivities. After 2 years of follow-up, no local recurrence was noted (Fig. 6) but the roots showed slightly signs of external resorption.

## Discussion

SBC is a benign intraosseous pseudocyst, most commonly observed in young adults. The mandible is almost exclusively affected. Most SBCs are discovered incidentally. Usually, SBC is radiolucent and unilocular with no or only slight expansion of bone and cortical thinning. Superior margins extend between the roots of teeth and are characteristically scalloped and corticated [11]. MRI demonstrates presence of fluid, fibrous connective tissue and/or gas. It confirms its cystic nature [12]. MRI features of SBC were highly characteristic and might allow them to be differentiated from others jaw diseases [13]. In this case, the combination of clinical and radiological findings confirmed the diagnosis of SBC. Given the benign nature of SBC and absence of epithelial lining, a minimally invasive approach through was warranted using a surgical guide for optimal bone cavity access. The objective of this surgical procedure was to achieve bone reossification while avoiding teeth and mandibular neurovascular bundle and ensuring minimal postoperative complications using a similar approach to guided targeted endodontic microsurgery [8, 14]. 

The main indications of 3D-printed surgical guide tools in oral and maxillofacial surgery include dental implant surgery, orthognathic surgery, mandibular reconstruction, Temporomandibular Joint reconstruction, maxillofacial prosthodontics and so far patient communication and education [15]. More recently, 3D-printed surgical guide tools were also used in the surgical management of jaws cysts and benign tumors [16, 17]. Two main types of surgical guide were designed: drilling location guides, which allows the surgical site to be precisely located, and osteotomy locating guides or cutting guides, which is used to delineate the osteotomy lines [16, 18–21]. Despite their benign nature, the excision of jaw cyst or tumoral lesions could require wide surgical access and extensive bone removal [22]. Management of less invasive pathology procedures might not require major reconstruction, however, use of surgical guide allows precise localization of the lesions with minimal bone removal, which is especially important when lesions are close to “noble organs” [23]. 

In a similar field, bone biopsies with 3D-printed surgical guide may be a helpful tool for enhancing the accuracy of the biopsy. Postl et al. demonstrated that guided procedures were more accurate with 3D-printed surgical guides compared to freehand methods. The mean deviation between biopsy axes was significantly lower in guided procedures than in freehand biopsies (1.4 mm ± 0.9 mm vs. 3.6 mm ± 1.0 mm; *p* = 0.0005). Similarly, the mean biopsy angle deviation was significantly lower in guided biopsies compared to freehand biopsies (6.8 ± 4.0 degrees vs. 15.4 ± 3.6 degrees; *p* = 0.0005). However, there was no significant difference in biopsy depth between guided and freehand biopsies [24]. 

Lotz et al. also described a tooth-supported drilling template technique for guided biopsy of the jawbone. They compared variation of digital planning of the biopsies and the position of the biopsies cylinder on the post-operative CBCT on fourteen patients. They found a mean angular deviation of 4.35 ± 2.5 degrees and a mean depth deviation of 1.40 ± 1.41 mm [17]. 

No clinical studies have so far been conducted on large patient samples to compare the accuracy or correspondence between the planned position in the software and the actual implant position post-intervention for traditional guided surgery systems versus the current sleeveless system.

Nevertheless, this sleeveless guided surgery system was tested, in order to determine the accuracy of implant insertion with one-piece ceramic implants. In total, 12 patients were enrolled in that study and installed with 20 implants by means of the aforementioned sleeveless static surgical guides. The accuracy of implant placement was checked using a non-invasive method, which permitted comparison of the planning data with the actual position of the fixtures after surgery. All implants were placed without any clinical problem and the mean deviations were 0.52 mm (95%CI: 0.37–0.67 mm) at the implant shoulder and 0.82 mm (95%CI: 0.56–1.08 mm) at the implant apex. Finally, the mean angular deviation was 2.85° (95%CI: 2.18°- 3.51°) with a deviation in height/depth of 0.35 mm (95%CI: 0.01–0.68 mm) [25]. Moreover, Zhao et al. (2023) showed in clinical endodontic microsurgery study that a designed 3D-printed surgical guide improve accuracy by fixing both the position and the angle of apectomy [26]. Ackerman et al. (2019) and Hawkins et al. (2020) also concluded, respectively in cadaver study and in vitro study, that targeted guided endodontic microsurgery was more accurate compared to freehand surgery to determine peri-apical lesion location and remove it, similarly as in our SBC case [8, 14]. 

The presence of two guides that drive the handpiece, positioned laterally, might potentially contribute to stabilizing trephine placement and reducing errors [25, 27, 28]. The access and visibility of the lesion facilitated the rapid curettage with safe excision of the ring bone without any induced forces this was reflected positively on the postoperative outcomes minimizing pain, swelling and trismus while, preserving the dental roots and the inferior alveolar nerve integrity. This kind of surgical guide can solves the lack of vertical space in mouth’s posterior region and allows the clinicians to work with shorter trephines or drills permitting to perform surgeries in partially dentate patients with limited mouth opening [28–30]. It was nevertheless necessary to evaluate the laxity of cheek musculature in pre-operative time.

The use of trephine drill-guided osteotomies can induce excessive frictional heat, leading to thermal damage of soft and hard tissue. The trephine has closely arranged cutting blades at the tip, which generate significant friction. Heat generation with trephines was found to be greater compared to conventional drills [31]. To counteract excessive heat generation, a large diameter and reduced depth penetration have shown lower heat generation during guided osteotomy preparation [32]. Moreover, the specific guide design allows permanent direct external irrigation at the trepanation site [25], with pumping movements limiting osseous necrosis [17]. 

A therapeutic alternative that could have been considered in our case is dynamic surgical navigation. This approach is comparable to static guided surgery in terms of accuracy, minimally invasive intervention, reduced operating time with minimal space requirement for drilling with a very good surgical open access [33, 34]. However, the necessity of a specific costly device, operator’s learning curve with specific ergonomics (indirect vision, size of the contra-angle) don’t allow dynamic navigation to be used by all surgeons [35, 36]. Similarly, augmented reality could be an other interesting approach to determine the better surgical access with a minimal invasiveness but need to be confirmed with further studies [37, 38]. 

Presurgical errors can manifest at various step of guide fabrication, including manual or digital impression-taking, cast fabrication, or registration of DICOM dataset with mouth impression STL dataset. Errors may also occur during surgery if the guide is not accurately placed, leading to tilting, or if sideway forces are applied during the ostectomy. Additionally, errors can happen if the drill tilts, particularly in cases where there is a small but specific space between the guide hole and the drill [39]. The fabrication of surgical guides is time-consuming and labor-intensive. Consequently, most dental office lack the capacity to produce in-house surgical guides. While external manufacturers can create case-specific surgical guides, the associated costs could be expensive [39, 40]. 

However, preoperative planning takes more time, is more difficult or requires a certain degree of knowledge and techniques in computer sciences. Surgical guides surely reduce operative time and reduce operating room costs secondary to shortening procedure times [41]. 

The use of a surgical guide greatly increases the effectiveness of a procedure. Surgical guides enhance the accuracy and predictability of the surgery, decrease technologic sensitivity and decrease the time required for surgery.

## Conclusion

In the procedure described in this report, the custom-made sleeveless static surgical guide played a key role in the success of the surgery because providing a precise location for the SBC. This allowed for a minimal window for accessing to the lesion and avoiding tooth and inferior alveolar bundle. As a result, a complete reossification was obtained with no surgical complications. It represents a surgical tool to determine the location and could be indicated in various cases cysts and tumors of the jaw.

## Data Availability

No datasets were generated or analysed during the current study.

## Abbreviations

CBCT: Cone beam computed tomography
MRI: Magnetic resonance imaging
SBC: Solitary bone cyst
STL: Stereolithographic
